# Tenofovir diphosphate levels in dried blood spots are associated with virologic failure and resistance to first‐line therapy in South Africa: a case–control cohort study

**DOI:** 10.1002/jia2.25849

**Published:** 2021-12-15

**Authors:** Jose R. Castillo‐Mancilla, Johnathan A. Edwards, Jaysingh Brijkumar, Mahomed‐Yunus Moosa, Yuan Zhao, Igho Ofotokun, Brent A. Johnson, Mitchell H. Lee, Selvan Pillay, Melendhran Pillay, Pravi Moodley, Daniel R. Kuritzkes, Henry Sunpath, Lane R. Bushman, Lucas Ellison, Peter L. Anderson, Vincent C. Marconi

**Affiliations:** ^1^ Division of Infectious Diseases Department of Medicine University of Colorado Anschutz Medical Campus Aurora Colorado USA; ^2^ Division of Infectious Diseases Department of Medicine Emory University School of Medicine Atlanta Georgia USA; ^3^ School of Health and Social Care University of Lincoln Lincoln UK; ^4^ National Health Laboratory Service Durban South Africa; ^5^ Department of Biostatistics and Computational Biology University of Rochester Rochester New York USA; ^6^ University of KwaZulu‐Natal Durban South Africa; ^7^ Divison of Infectious Diseases Brigham and Women's Hospital Harvard Medical School Boston Massachusetts USA; ^8^ Rollins School of Public Health Emory University Atlanta Georgia USA; ^9^ Emory Vaccine Center Emory University Atlanta Georgia USA

**Keywords:** adherence, dried blood spots, HIV resistance, South Africa, tenofovir diphosphate, viral failure

## Abstract

**Introduction:**

Tenofovir diphosphate (TFV‐DP) in dried blood spots (DBS), a measure of cumulative antiretroviral therapy (ART) adherence, is associated with viral suppression and predicts future viremia in persons with HIV (PWH). However, its utility to identify those at risk for virologic failure (VF) and drug resistance is unknown. To address this, we aimed to establish the association between this adherence biomarker and VF with drug resistance in a cohort of PWH initiating first‐line ART in KwaZulu‐Natal, South Africa.

**Methods:**

PWH initiating TFV disoproxil fumarate (TDF)‐based ART within a parent prospective cohort were evaluated. Using a nested design, DBS for TFV‐DP were collected from cases who developed VF (HIV‐1 RNA ≥1000 copies/ml) after ≥5 months on ART versus controls, matched 1:2 by site, age, gender, race and ART duration. Cases were categorized as having VF with or without resistance using genotyping. One‐way analysis of variance (ANOVA) was used to compare TFV‐DP for controls, cases with VF and resistance, and cases with VF without resistance. Data are presented as mean (standard deviation, SD) or geometric mean [95% confidence interval, 95% CI].

**Results and discussion:**

One thousand participants were enrolled in the parent study between 2014 and 2016, of which 288 (29%) had DBS available. Of these, 94 (33%) were cases and 194 (67%) were controls; 59% were women. Mean age of our population was 33 (SD 8) years. Genotyping was available in 50 (53%) of the 94 cases. Geometric mean TFV‐DP in DBS from controls was 708 [95% CI; 647–773] fmol/punch, which was higher compared to participants having VF with resistance (*n* = 36), 386 [95% CI; 241–617] fmol/punch and VF without resistance (*n* = 14), 61 [95% CI; 22–164] fmol/punch; *p*<0.001. Genotype could not be obtained in 44 (47%) cases.

**Conclusions:**

TFV‐DP in DBS showed a stepwise association with VF and drug resistance in South African PWH. Participants having VF with resistance had mid‐range concentrations of TFV‐DP, which were higher than those for PWH without resistance. Future research on the clinical utility of TFV‐DP concentrations in DBS to predict and prevent the development of VF and drug resistance is needed.

## 1 INTRODUCTION

Although current antiretroviral therapy (ART) is highly effective, durable adherence remains necessary to sustain viral suppression in persons with HIV (PWH). Unfortunately, up to 30–40% of PWH worldwide are not virologically suppressed [1, 2, 3], which limits the efficacy of treatment as prevention of HIV transmission [4]. While not observed in all cases of virologic failure (VF), the prevalence of drug resistance is highly variable, with rates as high as 54% for tenofovir and 82–92% for lamivudine (3TC) and efavirenz (EFV), respectively, in sub‐Saharan Africa [5, 6, 7]. An additional challenge is the limited availability of HIV‐1 RNA viral load (VL) and genotypic drug resistance testing in many countries in the region, making it difficult to identify PWH who require treatment modification to second‐line regimens due to drug resistance [5, 6].

Low ART adherence is a major driver of VF and drug resistance [8]. Despite its importance, accurately quantifying adherence has remained a challenge given the limitations of self‐report and other subjective measures [9]. Pharmacologic measures of short‐term adherence, such as antiretroviral concentrations in plasma, have been used to predict VF and drug resistance [10, 11, 12, 13]. However, they are limited by their qualitative nature in relation to dosing [14]. In contrast, pharmacologic measures of cumulative adherence (e.g. drug concentrations in hair or dried blood spots [DBS]) objectively quantify adherence and inform average dosing over the preceding weeks/months [9, 14]. Low drug concentrations in hair have previously predicted VF and drug resistance [15, 16, 17, 18], but collecting hair is still limited by availability and/or patient willingness to provide a hair sample [14]. Tenofovir diphosphate (TFV‐DP) in DBS quantifies cumulative adherence to tenofovir disoproxil fumarate (TDF) [19] or tenofovir alafenamide [20] and can be obtained during routine blood collection. This adherence biomarker is associated with viral suppression [21, 22], and predictive of future viremia, even among PWH who are virologically suppressed [23] both in the United States and South Africa. However, its ability to identify treatment failure (i.e. VF) with drug resistance is poorly understood [24].

To determine the association between cumulative ART adherence – quantified using TFV‐DP in DBS – and VF with or without drug resistance in South Africa, we evaluated this adherence biomarker within a prospective cohort study of PWH initiating first‐line ART in KwaZulu‐Natal (KZN).

## 2 METHODS

### 2.1 Study design

Treatment naïve PWH ≥18 years, who initiated first‐line ART according to the South African National HIV guidelines, were enrolled into the KZN HIV AIDS Drug Resistance Surveillance Study (ADReSS) [25]. ART consisting of TDF and emtricitabine (FTC), plus EFV (as a single‐table regimen) or nevirapine (NVP) [25] was provided by South African health authorities. PWH were excluded if they had received any prior ART (including pre‐ or post‐exposure prophylaxis), but single‐dose NVP for the prevention of mother‐to‐child transmission was permitted. Study participants were prospectively enrolled at two clinical centres, RK Khan (RKK) regional hospital and Bethesda (BTD) hospital in KZN, as previously described [25].

Enrolment initiated in July 2014 and was completed in September 2016. Baseline was considered the time of ART initiation and study enrolment. Study participants were followed per routine clinical standard of care, without any additional intervention. The study team assessed the health records and available data (including the National Health Laboratory System and the Department of Home Affairs data base) to determine the vital status of participants who were lost to follow‐up. Blood for VL at 6 and 12 months after ART initiation (followed by yearly intervals) was obtained per local standard of care [25] or until participants developed VF. Local guidelines defined VF as two consecutive HIV‐1 RNA >1000 copies/ml obtained ≥2 months apart after intensive adherence counselling. Using a nested case–control design, cases were defined as having only one HIV VL ≥1000 copies/ml after receiving ≥5 months of ART. Cases were matched 2:1 with controls (participants who had HIV VL <1000 copies/ml) by age, gender, race, duration of ART and site. Participants were blinded as to whether they were a case or a control until after they completed their follow‐up study visit surveys. Then, participants followed clinic protocol for adherence counselling and a subsequent confirmation VL. This procedure was intended to avoid any influence of adherence counselling on drug concentrations or survey responses. The observation and data collection period concluded in July 2018.

All patients provided informed written consent prior to their participation in the study. The study was approved by the University of KZN Biomedical Research Ethics Committee and Emory University Institutional Review Board.

### 2.2 Laboratory assessments

An ethylenediaminetetraacetic acid (EDTA) plasma sample from the study visit was used for the DBS, to quantify the VL, and for genotypic drug resistance testing on all cases. Sequencing was performed using a validated in‐house assay using primers adapted to subtype C [26]. Participants were defined as resistant if ≥1 major mutation in any drug class was identified using the Stanford database algorithm. Controls also had whole blood for DBS collected in EDTA tubes at the time of their last study visit. DBS were prepared by spotting 25 μl of whole blood onto Whatman 903 Protein Saver cards, which were allowed to dry for at least 2 hours (up to overnight) and frozen at –80°C until shipment [27, 28]. Drug concentration analysis was performed from a 3‐mm punch using a previously validated liquid chromatography/tandem mass spectrometry assay with a limit of quantification of 25 fmol/sample, for which stability data for long periods of time (e.g. >500 days) are available [28].

### 2.3 Statistical analysis

One‐way analysis of variance (ANOVA) was used to compare TFV‐DP in DBS at the time of the last study visit between cases and controls, who were divided into three groups: (1) controls; (2) cases having VF without resistance; and (3) cases having VF with resistance. Data were presented as *n* (%), mean (standard deviation, SD), median (interquartile range, IQR) or geometric mean [95% confidence interval, 95% CI]. Concentrations of TFV‐DP that were below the limit of quantification were imputed to 12.5 fmol/punch [21]. To allow for heteroscedastic errors, account for skewed drug concentrations, and facilitate the clinical interpretation of our findings, we used median regression to estimate differences in concentrations among the different groups. Standard errors for the median regression were computed using resampling procedures under the assumption that the errors are independent and identically distributed. Wald‐type confidence intervals were constructed at the nominal 5% level. Exact conditional logistic regression, stratified on matched case–control sets, was used to estimate the odds of viral failure for a 250 fmol/punch decrease in TFV‐DP (i.e. approximately a decrease in one TDF dose per week [27]). All statistical calculations were computed in the R statistical software (R Core Team, www.cran.r‐project.org).

## 3 RESULTS AND DISCUSSION

A total of 1000 treatment‐naïve PWH who initiated first‐line ART (TDF/FTC/EFV or TDF/FTC plus NVP [*n* = 6]) at RK K (*n* = 500) and BTD (*n* = 500) enrolled into the parent study [25]. For this analysis, we included data from 288 (29%) participants in whom drug concentrations were available (*n* = 1 on NVP). Of these 288, 194 (67%) were controls and 94 (33%) were cases. The mean age for the analysed cohort was 33 (SD 8) years and was similar between cases and controls (Table 1). Additional demographic and baseline clinical characteristics of the study participants are shown in Table 1. Overall, 169 (59%) participants included in this analysis were women, and 96% were Black. The overall median duration of ART was 35 (IQR; 31–42) weeks for cases and 34 (IQR; 27–42) days for controls (Table 1). Cases had a higher median VL at the time of case/control determination (≥5 months on ART) as compared to controls, 10,600 (IQR; 20–1,060,000) copies/ml versus 40 (IQR; 20–544,000) copies/ml (Table 1). The median CD4^+^ T cells at the time of case/control determination (≥5 months on ART) was 301 (IQR; 10–988) cells/μl for controls versus 259 (IQR; 4–687) cells/μl for cases.

**Table 1:** Patient characteristics stratified by virologic failure status (cases vs. controls)

	Case (*n* = 94)	Control (*n* = 194)	Overall (*n* = 288)	*p* Value
Age at first visit (years), mean (SD)	33 (9)	33 (8)	33 (8)	0.67
Gender, *n* (%)				1.00
Female	54 (57%)	115 (59%)	169 (59%)	–
Male	40 (43%)	79 (41%)	119 (41%)	–
Race, *n* (%)				0.20
Black	86 (92%)	190 (98%)	276 (96%)	–
Indian	6 (6%)	4 (2%)	10 (4%)	–
Coloured	2 (2%)	–	2 (0.7%)	–
Ethnicity or nationality, *n* (%)				0.20
Zulu	67 (71%)	156 (80%)	223 (77%)	–
Xhosa	11 (12%)	22 (11%)	33 (12%)	–
Other	10 (11%)	12 (6%)	22 (8%)	–
Missing	6 (6%)	4 (2%)	10 (4%)	–
Viral load at the time of case/control determination^a^ (copies/ml), median (IQR)	10,600 (20–1,060,000)	40 (20–54,400)	43 [20–1,060,000]	<0.001
Missing	1 (1%)	–	1 (0.3%)	–
Absolute CD4^+^ T‐cell count at baseline (cells/mm^3^), median (IQR)	259 (4–687)	301 (10–988)	287 (4–988)	0.06
Time on ART at the time of DBS sampling (weeks), median (IQR)	35 (31–42)	34 (27–42)	–	0.6
VF unable to genotype (*N* = 44)	37 (33–42)	–	–	
VF with resistance (*N* = 36)	33 (29–41)	–	–	
VF without resistance (*N* = 14)	34 (32–44)	–	–	
Viral failure status, *n* (%)				NA
VF unable to genotype (*N* = 44)	44 (47%)	–	44 (15%)	–
VF with resistance (*N* = 36)	36 (38%)	–	36 (13%)	–
VF without resistance (*N* = 14)	14 (15%)	–	14 (5%)	–
Single dose of nevirapine, *n* (%)				1.00
Yes	7 (7%)	18 (9%)	25 (9%)	–
No	87 (93%)	176 (91%)	263 (91%)	–
NRTI mutations, *n* (%)				NA
Yes	29 (31%)	–	29 (10%)	
No	20 (21%)	–	20 (7%)	
Missing	45 (48%)	194 (100%)	239 (83%)	
NNRTI mutations, *n* (%)				NA
Yes	36 (38%)	–	36 (13%)	
No	13 (14%)	–	13 (5%)	
Missing	45 (48%)	194 (100%)	239 (83%)	

Note: Data are presented as mean (SD), *n* (%) or median (IQR). *p* values compare cases (PWH with virologic failure) and controls (PWH without virologic failure), calculated using *t* tests for numeric variables and χ^2^ tests for categorical variables.

^a^ Occurred ≥5 months after ART initiation.

Abbreviations: DBS, dried blood spots; NA, not available; NNRTI, non‐nucleoside reverse transcriptase inhibitor; NRTI, nucleoside reverse transcriptase inhibitor; VF, virologic failure.

Genotyping was successfully obtained for 50 (53%) cases, 36 (38%) of whom had evidence of drug resistance (Table 1). Among these, 29 (31%) participants had NRTI‐associated mutations, while 36 (38%) had NNRTI‐associated mutations (Table 1). For 44 (47%) cases, the VL was too low to genotype, sequence had poor quality or the sample was unavailable (median HIV VL [IQR] was 133 [20–741] copies/ml, data missing for four cases).

**Figure 1:**
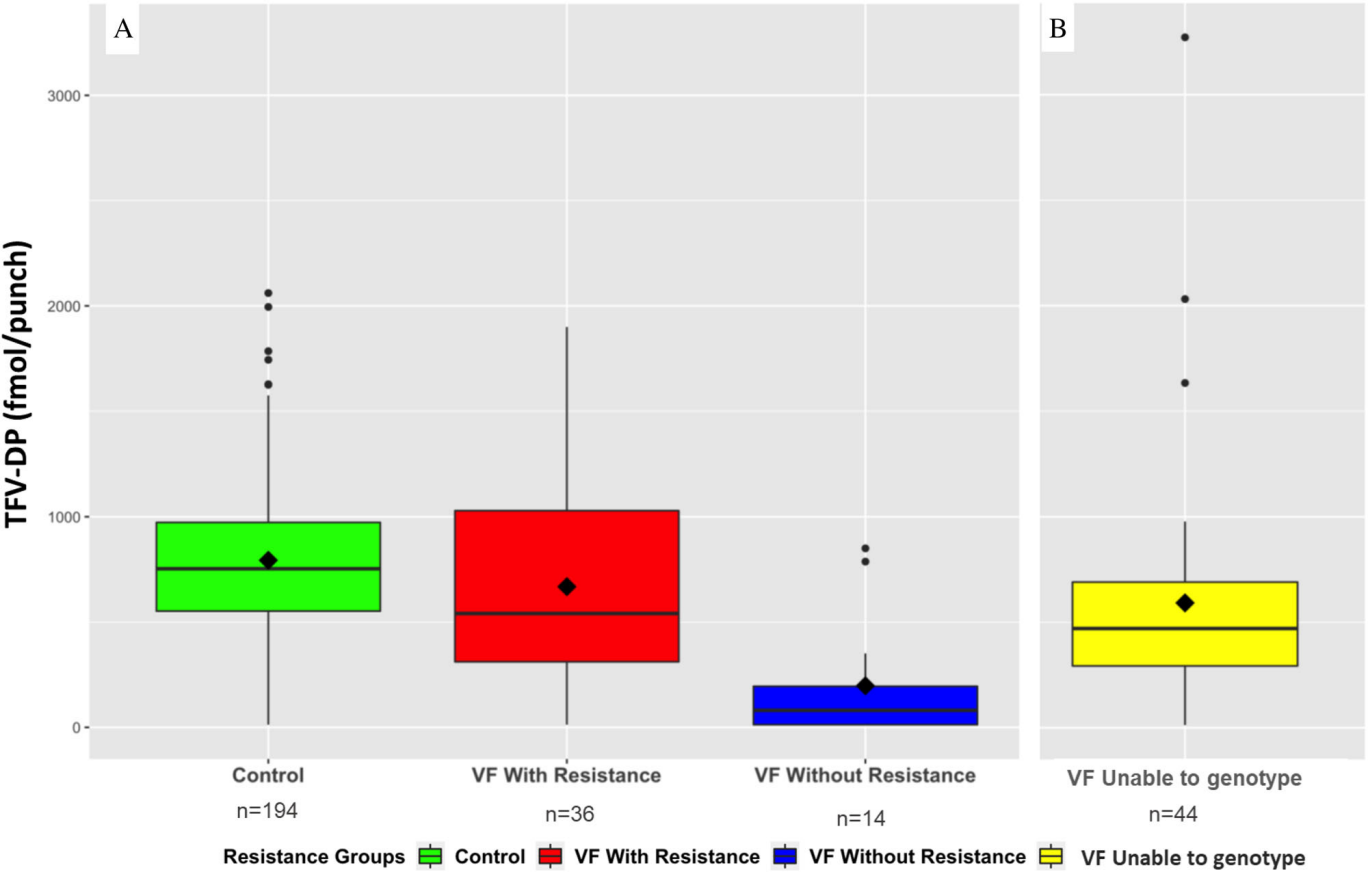
Concentrations of tenofovir diphosphate in dried blood spots in controls and cases according to their resistance testing status (*n* = 288). Panel a shows controls and cases with and without resistance (*n* = 244). Panel b shows cases in whom genotyping could not be performed due to a low HIV VL, missing sample or poor quality sequence (*n* = 44). Boxes represent median (interquartile range) TFV‐DP concentrations in DBS. Diamonds represent geometric mean TFV‐DP concentrations in DBS. Whiskers represent approximate 95% confidence intervals for normally distributed data. Abbreviations: DBS, dried blood spots; TFV‐DP, tenofovir diphosphate; VF, virologic failure.

DBS for TFV‐DP were available for analysis in 288 participants (194 controls and 94 cases). Table 2A shows the geometric mean [95% CI] concentrations of TFV‐DP in DBS in controls (*n* = 194), and in cases having VF with (*n* = 36) and without resistance (*n* = 14). Overall, there was a statistically significant gradual decline in TFV‐DP concentrations in DBS among each group (ANOVA *p*<0.001), with controls having the highest drug concentrations and cases without resistance exhibiting the lowest concentrations (708 [95% CI; 647–773] vs. 61 [95% CI; 22–164] fmol/punch, Table 2A and Figure 1). The geometric mean concentration of TFV‐DP among participants in whom genotyping was unsuccessful (*n* = 44) was 365 [95% CI; 249–535] fmol/punch (Figure 1).

**Table 2:** (A) Concentrations and (B) median differences of tenofovir diphosphate in dried blood spots at the time of last study visit in controls and cases according to their resistance testing status (*n* = 288)

A			
Category (*n*)	TFV‐DP in DBSGeometric mean(fmol/punch)	95%Confidence interval	*p* Value
Control (194)	708	(647–773)	<0.001
VF with resistance (36)	386	(241–617)	
VF without resistance (14)	61	(22–164)	
VF unable to genotype (44)	365	(249–535)	

B			
Category (*n*)	Median difference inTFV‐DP in DBS(fmol/punch)	95%Confidence interval	*p* Value
Controls (194)^a^	*REF*	*REF*	*–*
VF with resistance (36)	–202	(–343 to –61)	0.005
VF without resistance (14)	–604	(–819 to –389)	<0.001
VF unable to genotype (44)	–271	(–401 to –141)	<0.001

Note: *p*‐value compares all groups overall and was calculated using one‐way ANOVA.

^a^ Reference group were controls, with a median (95% confidence interval) concentration of TFV‐DP in DBS of 753 (697–808) fmol/punch.

Abbreviations: DBS, dried blood spots; TFV‐DP, tenofovir diphosphate; VF, virologic failure.

Using the control group as reference (median TFV‐DP 753 [95% CI; 697–808] fmol/punch), cases having VF with resistance had a median concentration of TFV‐DP in DBS that was 202 [95% CI; –343 to –61] fmol/punch lower than the controls (*p* = 0.005, Table 2B). In comparison, cases having VF without resistance and those in whom genotyping was unsuccessful had a median concentration of TFV‐DP in DBS that was 604 [95% CI; –819 to –389; *p*<0.001] and 271 [95% CI; –401 to –141; *p*<0.001] fmol/punch lower than the controls, respectively (Table 2B). The odds ratio comparing the odds of VF for a 250 fmol/punch decrease of TFV‐DP was statistically significant in the matched study sample (VF 1.48; 95% CI 1.22–1.78; *p*<0.001) and by genotype subgroup (VF with resistance 1.28; 95% CI 1.00–1.64; *p* = 0.049 and VF without resistance 4.49; 95 CI 1.23–16.4; *p* = 0.023).

In this study, we identified a stepwise decrease in TFV‐DP across categories of VF (with or without resistance) for South African PWH on TDF‐based ART. TFV‐DP concentrations in participants having VF with resistance were approximately 50% lower when compared to controls, while concentrations in participants having VF without resistance were very low (∼90% lower than controls), consistent with near‐complete non‐adherence to ART. This suggests that, while not enough to achieve suppression, mid‐range cumulative ART adherence is required to select for drug resistance, and that resistance is unlikely to develop in the absence significant cumulative drug exposure. Furthermore, we identified a four‐fold increase in the odds of VF without resistance for every decrease in one TDF dose/week in cases compared to controls. To our knowledge, this is the first report where TFV‐DP in DBS has been associated with antiretroviral drug resistance in South Africa.

Although the concentrations of TFV‐DP in virologically suppressed PWH in our study were lower than previously observed in the United States [21], they are consistent with other studies in Africa [22, 29, 30]. Our findings are also consistent with a clinical cohort of PWH in the United States, where TFV‐DP concentrations in study participants who developed new drug resistance‐associated mutations were ∼40% lower than those observed in participants who were virologically suppressed [21, 24]. These results are also similar to other studies that assessed ART adherence using qualitative [31, 32] and other cumulative [15, 33] pharmacologic measures. For studies where quantitative measures of cumulative ART adherence were used, an analysis of young PWH in Tanzania demonstrated that participants who failed EFV and NVP treatment without resistance had hair drug concentrations below the limit of detection [15]. However, this study did not assess the emergence of drug resistance‐associated mutations over time. By comparison, a study of 287 adult PWH receiving NNRTI or protease inhibitor‐based therapy in China identified a stepwise decline in 3TC hair concentrations, where mean 3TC concentrations in PWH who developed VF with drug resistance were 30% lower when compared to those who were virologically suppressed, but higher when compared to PWH who developed VF without resistance [33]. Collectively, these studies support the notion that pharmacologic measures of cumulative adherence can identify PWH on ART with partial non‐adherence – which is required to develop drug resistance – and distinguish them from those who are completely non‐adherent, in whom drug resistance is unlikely.

Our findings offer several potential clinical applications. First, when paired with HIV VL, TFV‐DP in DBS can provide a more comprehensive depiction of the adherence–VL relationship and inform the decision to obtain drug resistance testing if concentrations are mid‐range in the setting of viremia. Second, if genotyping is not widely available, a point‐of‐care assay for TFV‐DP [34] could identify PWH with VF who may have already developed drug resistance and facilitate a transition to second‐line regimens without the need for resistance testing. Third, since TFV‐DP in DBS can predict future viremia [23, 29, 30], this pharmacologic measure could help identify PWH at risk of VF in whom an adherence intervention is indicated. Future studies that can establish clinically relevant drug concentration thresholds and evaluate the clinical utility of prospectively assessing paired VL and TFV‐DP in DBS are required.

Strengths of our study include a large cohort of unselected PWH with a matched case–control study design and a large proportion of women (59%) receiving care in rural and peri‐urban clinics from a high HIV prevalence region in South Africa. This allows for the generalizability of our findings in these settings. We also evaluated a quantitative pharmacologic measure of cumulative adherence in a matrix (i.e. blood) that is collected as part of routine clinical care and has been studied in PWH from Africa [22, 29, 30]. Limitations include that we were unable to obtain genotypic resistance data in 44 participants due to a low VL or poor sequencing. This was likely due to an improvement in adherence between visits (i.e. median HIV VL at this visit was <200 copies/ml). Second, our DBS sampling strategy focused on the study visits when VF was documented and did not include timepoints that preceded this outcome, thus the duration of VF and drug resistance was not known. This could have limited our understanding of the utility of drug concentrations to prevent VF. Third, we focused on PWH taking fixed‐dose combination TDF/FTC/EFV, which limited our ability to assess selective non‐adherence when individual antiretrovirals are used. Also, our study was limited to NNRTI‐based therapy, which has a different genetic barrier and adherence–resistance relationship that protease and integrase inhibitors [35]. Last, we did not include an evaluation of emtricitabine triphosphate (FTC‐TP) in DBS, a measure of recent dosing, which has also been associated with viral suppression [36]. Future studies that assess whether this anabolite is also associated with drug resistance are required.

## 4 CONCLUSIONS

This study established a stepwise association between concentrations of TFV‐DP in DBS (a measure of cumulative ART adherence) and VF with and without resistance in PWH on TDF‐based therapy in South Africa. Drug concentrations in PWH who developed VF with drug resistance were approximately half those of PWH who remained virally suppressed and considerably higher than PWH who developed VF without resistance. Future studies are required to determine whether TFV‐DP in DBS can be used prospectively to monitor ART adherence and prevent VF and resistance, or whether it can be used in lieu of drug resistance testing to determine the need for regimen modification for PWH failing first‐line TDF‐based ART.

## COMPETING INTERESTS

JCM reports grant support from the National Institutes of Health/National Institute of Allergy and Infectious Diseases. VCM has received investigator‐initiated research grants (to the institution) and consultation fees (both unrelated to the current work) from Eli Lilly, Bayer, Gilead Sciences and ViiV. DRK serves as a consultant to and/or has received grant support from Gilead, GlaxoSmithKline, Merck and ViiV. PLA received personal fees and research funding paid to his institution from Gilead Sciences. All other authors declare no competing interests.

## AUTHORS’ CONTRIBUTIONS

JB, BJ, JAE, SP, MP, M‐Y M, DRK, IO, HS and VCM contributed to the cohort conception and design. DRK and VCM obtained the funding. JB, SP, MP, PM, M‐Y M, HS and VCM contributed to data collection. JCM, LRB, LE and PLA performed the drug concentration analysis. JCM, YZ, BJ, JAE, ML, DRK, PLA and VCM contributed to data analysis and interpretation. JCM wrote the first draft and subsequent revisions. All authors contributed to additional drafts, revisions and approved the final manuscript.

## FUNDING

Research reported in this publication was supported by the National Institute of Allergy and Infectious Diseases of the National Institutes of Health under Award Number R01AI098558 and R01AI138801. VCM received support from the Emory CFAR (P30AI050409). The content is solely the responsibility of the authors and does not necessarily represent the official views of the National Institutes of Health.

## PRIOR PRESENTATION OF STUDY RESULTS

This work was presented, in part, at the Conference on Retroviruses and Opportunistic Infections 2020, March 8–11, 2020, Boston, Massachusetts, Abstract 520.

## Data Availability

The data that support the findings of this study are available on request from the corresponding author. The data are not publicly available due to privacy or ethical restrictions.
